# A new protocol for absolute quantification of haemosporidian parasites in raptors and comparison with current assays

**DOI:** 10.1186/s13071-020-04195-y

**Published:** 2020-07-17

**Authors:** Xi Huang, Di Huang, Yuge Liang, Linlin Zhang, Guocheng Yang, Boye Liu, Yangyang Peng, Wenhong Deng, Lu Dong

**Affiliations:** 1 College of Life Sciences, Beijing Normal University, Beijing, China; 2 MOE Key Laboratory for Biodiversity Sciences and Ecological Engineering, Beijing Normal University, Beijing, China; 3 Shaanxi Institute of Zoology, Xi’an, China

**Keywords:** Avian haemosporidia, ddPCR, qPCR, Infection intensity, Raptor

## Abstract

**Background:**

Accurate quantification of infection intensity is essential to estimate infection patterns of avian haemosporidian parasites in order to understand the evolution of host-parasite associations. Traditional microscopy is cost-effective but requires high-quality blood smears and considerable experience, while the widely used semi-quantitative qPCR methods are mostly employed with ideal, laboratory-based golden samples and standard curves, which may limit the comparison of parasitemia from different laboratories.

**Methods:**

Here we present a digital droplet PCR (ddPCR) protocol for absolute quantification of avian haemosporidians in raptors. Novel primers were designed that target a conserved fragment of a rRNA region of the mitochondrial genome of the parasites. Sensitivity and repeatability were evaluated compared to qPCR and other assays.

**Results:**

This ddPCR assay enables accurate quantification of haemosporidian parasites belonging to *Plasmodium*, *Haemoproteus* and *Leucocytozoon* with minimum infection quantities of 10^−5^ (i.e. one parasite copy in 10^5^ host genomes) without the use of standard curves. Quantities assessed by ddPCR were more accurate than qPCR using the same primers through reduction of non-specific amplification in low-intensity samples. The ddPCR technique was more consistent among technical duplicates and reactions, especially when infection intensities were low, and this technique demonstrated equal sensitivity with high correspondence (*R*^2^ = 0.97) compared to the widely used qPCR assay. Both ddPCR and qPCR identified more positive samples than the standard nested PCR protocol for the *cytb* gene used for barcoding avian haemosporidians.

**Conclusions:**

We developed a novel ddPCR assay enabling accurate quantification of avian haemosporidians without golden samples or standard curves. This assay can be used as a robust method for investigating infection patterns in different host-parasite assemblages and can facilitate the comparison of results from different laboratories.
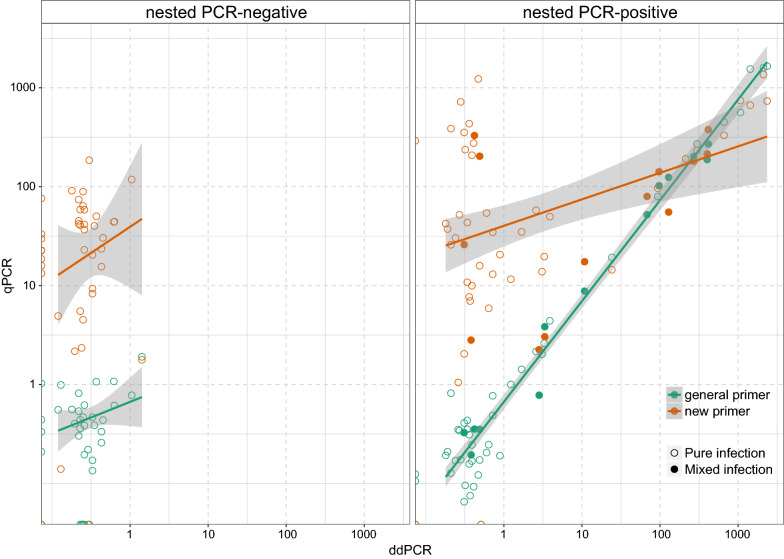

## Background

Avian malaria (*Plasmodium*) and related parasites (*Leucocytozoon* and *Haemoproteus*), also known as avian haemosporidians, are a diverse group of vector-borne blood parasites. They are responsible for infectious disease and accelerated senescence [1] in their bird hosts, and therefore, have gained extensive attention for decades, especially in regard to variations in host-parasite assemblages, which is of great importance to the understanding of disease evolution. Current identification of avian haemosporidians is mainly based on analysing blood samples from bird hosts [2]. Morphological and molecular methods have identified approximately 250 morphological species and over 3000 distinct *cytb* lineages, respectively [3], in thousands of bird species all over the world [4].

Assemblages of parasites and their hosts, estimated by prevalence and infection intensity, can vary both temporally and spatially due to complex and multidimensional environmental conditions [5], as well as host resistance to parasites [6], parasite adaptation to certain host species [7], and whether the host is harbouring two or more different parasites at the same time [8]. When analysing prevalence and infection intensity of parasites, variations in identification accuracy may lead to biases across studies, further inaccurate estimation of global patterns of host-parasite interactions, and may also lead to biases on how parasites adapt to certain host species under forces of natural selection. Therefore, accurate estimation of prevalence and infection intensity based on optimized methods are of urgent need in ecological and evolutionary studies of avian haemosporidian parasites [9].

A variety of studies have been carried out on the prevalence of avian haemosporidian parasites in natural communities and variations in relation to a set of environmental factors [10]. However, studies on infection intensity are still limiting despite the importance in reflecting adaptation of avian haemosporidian parasites, especially in generalist parasites with multiple host species [7]. Due to differences in host immune systems and host-parasite co-evolutionary history, infection intensity of a given parasite can differ dramatically among different hosts [11]. Occasionally, a parasite may encounter a host species to which it is not optimally adapted and thus fail to form gametocytes for secondary transmission [12]. As the PCR-positive results for avian haemosporidians cannot provide information on life stages of parasites, such abortive infections are difficult to identify but may be detected by quantification of a very low intensity infection. To further understand host specificity and the evolution of host-parasite associations, accurate quantification of infection intensities is essential.

The traditional light microscopy method can quantify infection intensity by counting infected red blood cells in blood smears, which is a cost-effective and straightforward method. However, this technique requires high-quality blood smears that are difficult to achieve in the field [13], and the false-negative ratio may increase if the infected individual carries parasite densities below the limit of detection by microscopy. A real-time quantitative PCR (qPCR) method [14] has been shown to be useful at assessing infection intensity in a variety of haemosporidian parasites and has also been shown to be more sensitive than microscopy [15], requiring less effort for sample preparation and technician experience. However, qPCR has been difficult to standardize and relies heavily on laboratory-based standard samples of absolute known parasite DNA concentrations, and this limits the comparison of results reported from different research laboratories. The recently developed digital-droplet PCR (ddPCR) method has been successfully employed for quantification of unicellular green algae [16], the protozoan parasite *Cryptosporidium* [17], cancer cells [18], and for human malaria [19] based on its high sensitivity and more reproducible measurements without standard samples compared to more traditional qPCR methods. Based on water-oil emulsion droplet technology, the entire ddPCR reaction system can be divided into approximately 20,000 nanolitre-sized droplets in random fashion for independent amplifications. Positive or negative amplification in each individual droplet is revealed by fluorescence detection, and the absolute copy number of the target gene in the original sample can be calculated using Poisson statistics [20]. Since PCR reactions in all droplets occur independently, various factors affecting the success of PCR can be diluted when analysing the combined results, including amplification efficiency, background DNA and other inhibitors, and the consumption of enzymes. As a result, inter-reaction variations can be largely reduced compared to other PCR methods, especially when the proportion of the target gene is small within the template, as is often the case in avian haemosporidian parasites in blood samples.

Given this, a ddPCR method can be an ideal tool for absolute quantification of avian haemosporidians and can be used to further investigate infection patterns in different host-parasite assemblages. Although a ddPCR assay for human malaria has been developed [19] and widely applied for diagnostics of *P. falciparum* and *P. vivax*, the deep divergence of avian haemosporidian parasites compared to those that cause human malaria, along with the nucleate erythrocytes in birds that may induce non-specific amplification of host genomes, make this particular method unavailable for use in birds. Further, the widely used qPCR primer pair for avian haemosporidians [21] cannot be employed in ddPCR, due to its longer amplicon length (188 bp) than that required for ddPCR (around 80–130 bp).

To enable more accurate and comparable quantification of the infection intensity of haemosporidian parasites, we developed a novel ddPCR assay test using 100 raptor samples in order to determine the following: (i) the sensitivity and repeatability of this novel ddPCR protocol; (ii) the accuracy in diagnostics of haemosporidian parasites and quantification of infection intensity compared to nested PCR and qPCR; and (iii) whether measurements in haemosporidian detection are related to different parasite genera or mixed infections.

## Methods

### Sample collection and parasite identification

Blood samples (*n* = 100) of 15 raptor species (Additional file 1: Table S1) were collected at the Beijing Raptor Rescue Centre between 2016 and 2017 and stored in absolute ethanol. Two blood slides were made for 93% of the samples and inspected under an optical microscope (Olympus, Tokyo, Japan) for determination of haemosporidian infection and infection intensity. Blood smears were first examined at a medium magnification (400×), and then with at least 20 fields at a high magnification (1000×) with oil immersion [22].

DNA was extracted using a TIANamp DNA kit (Tiangen Biotech Ltd., Beijing, China) according to the manufacturer’s protocol and diluted to 20–30 ng/µl for further analysis. Molecular identification was carried out following a nested PCR protocol [23] to amplify the partial *cytb* gene of avian haemosporidian parasites. Either a 479-bp fragment of *Plasmodium* and *Haemoproteus* parasites (HaemF/HaemR2) or a 480-bp fragment for *Leucocytozoon* (HaemFL/HaemR2L) was amplified. Positive samples were determined by the presence of bands at the target location on a 2% agarose gel and were sequenced from both ends using a 3730XL automatic sequencer (Applied Biosystems, Foster City, California, USA). All samples were sequenced at least twice for both primer pairs to check for possible mixed infection or false positives. Obtained sequences were compared with those compiled in the MalAvi database [3] for taxonomic identification. Parasites with at least one base-pair difference to *cytb* sequences in MalAvi were defined as novel lineages.

### ddPCR primer design and screening

To design primers for haemosporidian quantification, five whole mitochondrial genomes of three avian haemosporidian genera, *Haemoproteus* (*Parahaemoproteus*), *Leucocytozoon*, and *Plasmodium*, were obtained from GenBank (accession numbers CM004177.1, NC_015304.1, AB250690.1, AB250415.1 and LN835311.1) and aligned in Geneious v 11.0.5 (Biomatters Limited, Auckland, New Zealand). Based on the homologous sequences among different genera, a total of three primer pairs were designed using Primer3 in Geneious with default settings, targeting a 100–150 bp fragment in highly conserved regions. Primers were named according to their positions on the sequence of *Haemoproteus tartakovskyi* strain SISKIN1 (GenBank: CM004177.1). Seven samples infected by different lineages with high to low infection intensity were selected for screening the amplification efficiency and specificity of the candidate primers. Standard PCR reactions were conducted for each primer set and visualised *via* 2% agarose gel. The primer pair 3524F (5′-AGG CAA AGA AAA TGA CCG G-3′) and 3655R (5′-ATG GCG AGA AGG GAA GTG TG-3′), targeting a 131-bp fragment (excluding primers), was selected for further analysis based on its high efficiency and specificity to amplify the lineages of all three genera (Additional file 2: Figure S1). The other primer pairs were excluded in further analyses due to either low amplification efficiency or low sensitivity.

### Quantification of parasite intensity by ddPCR

The ddPCR reaction contains 2 µl of DNA template, 0.5 µM each of forward (3524F) and reverse (3655R) primers, 10 µl of EvaGreen Supermix (Bio-Rad, Hercules, California, USA), and ddH_2_O to reach a total volume of 20 µl. The reaction was transferred to an 8-well cartridge to mix with 70 µl Droplet Generation Oil for EvaGreen (Bio-Rad), and a QX200TM Droplet Generator (Bio-Rad) was used to generate droplets. The mixture was then transferred to a 96-well PCR plate for PCR amplification on a C1000 Touch TM (Bio-Rad) instrument, starting with 5 min incubation at 94 °C, followed by 35 cycles (30 s at 94 °C, 30 s at 57 °C, and 40 s at 72 °C) and a final 10 min extension at 72 °C. All samples were run in triplicate, and each plate contained at least three non-template controls (NTCs) to detect false positives and were adjusted for the threshold of positive detection. After the PCR reaction, the whole plate was loaded on a Bio-Rad QX200TM Droplet Reader for positive and negative droplet detection using the absolute quantification (ABS) method and analysed using QuantaSoft^TM^ software (Bio-Rad). Threshold values were set automatically to generate the quantity of target gene (copies/µl) in each sample. Patterns of droplet distribution were inspected to ensure that the droplets in NTCs were all below threshold, and false positives were identified according to the “rain” function (Additional file 3: Figure S2). The DNA concentration of all positive samples was assessed on a Qubit 3.0 Fluorometer (Invitrogen, Carlsbad, California, USA) using the dsDNA BR Assay Kit (Invitrogen).

### Quantification of parasite intensity by qPCR

To compare the efficiency and accuracy of the new and widely used primers in real-time qPCR for avian haemosporidian parasites, two qPCR reactions were carried out in parallel using the widely applied primers 343F-496R (hereafter general qPCR), which amplify a partial mitochondrial rRNA gene [21], and the novel primers 3524F-3655R (new primer qPCR). All qPCR reactions were performed on a 7500 Real-Time PCR instrument (Applied Biosystems) using a TB Green Premix reaction kit (Takara Bio Inc., Shiga, Japan). All samples were run in duplicate, together with two NTCs to detect false positives.

Each of the 20 µl qPCR reactions included 2 µl of DNA template, 0.8 µl of each primer (10 ng/µl), 10 µl TB Green Premix buffer, 0.4 µl Rox-dye, and 6 µl ddH_2_O. After 30 s incubation at 95 °C, the amplification steps (5 s at 95 °C, 34 s at 52 °C for 343F-496R and 57 °C for 3524F-3655R, 30 s at 72 °C) were run for 40 cycles, immediately followed by a melting analysis between 60 °C and 95 °C. After each reaction, the Cq value (i.e. the cycle during which the fluorescence signal reached threshold) of each sample was obtained from the amplification curve and scored as the average of the duplicates. The result was accepted only when the fluorescence signal in NTCs was below the threshold. Melting curves were inspected to check for false positives, i.e. a Cq value was generated but the melting peak corresponded to non-specific amplifications (Additional file 4: Figure S3). To check and adjust variations between qPCR reactions, three samples with high to low infection intensities (160520: 7.02%; 160514: 0.9%; 160608: 0.4%) were selected as “golden standard samples” [7] and included in all reactions. To obtain the amplification efficiency in qPCR analyses, each of the golden samples were 4-fold serially diluted to five gradients to generate the standard curves and were equally distributed to several isolated tubes for different PCR reactions to avoid cross-contamination. The amplification efficiency (Eff) in each reaction was calculated based on the Cq values of the serially-diluted samples using linear regression, and parasite quantities in positive samples (x) were calculated using the ΔCq method: ${\text{Qx}} = {\text{Qgs}} \times \left( {1 + Eff} \right)^{{\left( {Cqgs - Cqx} \right)}}$.

### Data analysis

Assessed parasite quantities (both by ddPCR and qPCR) were divided by host quantities in the corresponding sample to obtain infection intensities. As in the extracted DNA samples, the vast majority should correspond to the host genomic DNA, while only a tiny proportion (< 0.02%) from the parasite [24]. Host quantities (in terms of copies/µl) were calculated based on the genome weight (acquired from the Animal Genome Size Database, www.genomesize.com) of certain raptor species (Gh) and the initial DNA concentration assessed using a Qubit 3.0 fluorometer (Invitrogen). In other words, the infection intensity in sample x was calculated according to the formula $${\text{Inf}} = {\raise0.7ex\hbox{${Qx}$} \!\mathord{\left/ {\vphantom {{Qx} {Gh}}}\right.\kern-0pt} \!\lower0.7ex\hbox{${Gh}$}} \times Conc$$, where Qx for ddPCR was obtained automatically and calculated as described above for qPCR.

A linear regression was carried out to test the consistency of parasite quantities assessed by ddPCR and qPCR methods using the same primer pair, while the repeatability of technical replicates (*rpt*) was estimated for each method using the *rptR* package [25]. Quantification results of ddPCR and qPCR using the newly-developed primer set were compared using a paired-samples t-test in the high parasite quantity group (> 1 copy/μl) and the low quantity group (< 1 copy/μl) to evaluate the consistency of the two methods in the scenario of different infection intensity levels. In all reactions, samples were considered positive even if only one of the technical replicates appeared to be successfully amplified.

In order to investigate whether the detection success of ddPCR was related to haemosporidian genera or mixed infections, ANOVAs were used to test variations in haemosporidian quantities in different samples. All data were analysed in R version 3.5.3 [26].

## Results

### Parasite identification and robustness of ddPCR

Among the 100 samples tested by nested PCR, 42 were negative (nested PCR-negative) and 58 were positive (nested PCR-positive), of which 51 were successfully sequenced. A total of 34 distinct lineages (7 previously recorded lineages and 27 novel) were identified, including 8 *Plasmodium* lineages, 15 *Haemoproteus* and 11 *Leucocytozoon* (Additional file 1: Table S1) lineages. More than one haemosporidian lineage was detected in 11 samples. These were defined as mixed infections, mostly comprising *Haemoproteus* + *Leucocytozoon* (Additional file 1: Table S1). All sequences were uploaded to GenBank under the accession numbers MT281461, MT281462, MT281464-MT281466, MT281474-MT281479, MT281481-MT281484, MT281489, MT281494, MT281499, MT281500, MT281502-MT281506, MT281512, MT281514, MT281516-MT281518, MT281521, MT281522 and MT281526.

**Fig. 1:**
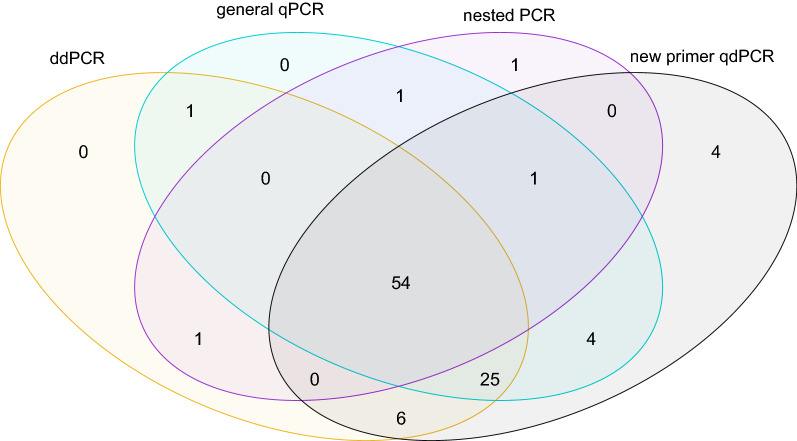
Comparison among the four methods in identification of positive samples of haemosporidian infections in raptors

All positive samples identified by ddPCR and general qPCR were confirmed by at least one another diagnostic method (Fig. 1), and both techniques resulted in higher prevalence estimates (ddPCR: 87%; general qPCR: 86%) than nested PCR (58%) and light microscopy (Additional file 1: Table S1). The nested PCR-negative samples had very low infection quantities in both ddPCR (mean: 0.35 copies/μl, 95% CI: 0.26–0.45) and general qPCR (mean: 0.55 copies/μl, 95% CI: 0.41–0.69), whereas the nested PCR-positive samples had significantly higher quantities (ddPCR: mean: 176.13 copies/μl, 95% CI: 46.03–306.23; general qPCR: mean: 131.12 copies/μl, 95% CI: 32.06–230.18), which implies the false-negative results from nested PCR were mainly due to a very low density of parasites. Differences were not affected when lineages of different haemosporidian genera (*F*_(6, 79)_ = 0.41, *P* = 0.86) were considered or when the sample carried a mixed infection (*F*_(1, 84)_ = 0.27, *P* = 0.61).

**Fig. 2:**
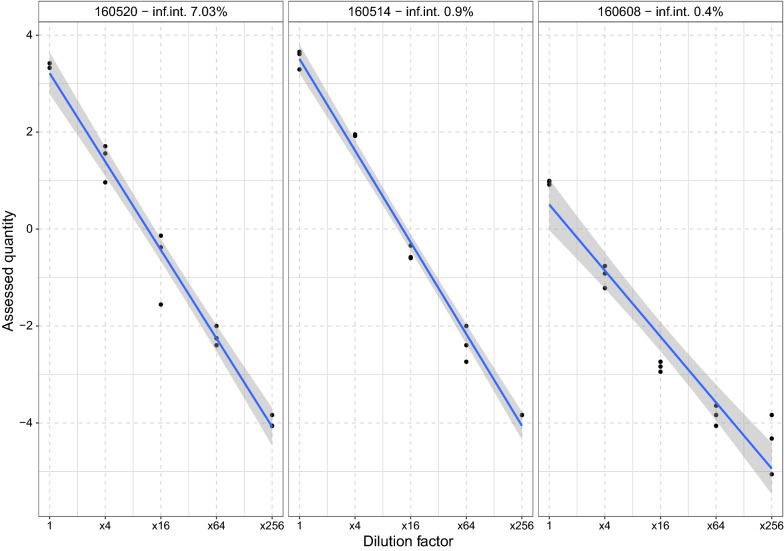
Quantification of three sets of serially-diluted samples with different infection intensities by ddPCR. *Note*: the y-axis was log_10_-converted

ddPCR showed consistency across the dilution gradient in high-intensity samples (*β* = 0.97 ± 0.06, *R*^2^ = 0.98, *P* < 0.001) and medium ones (*β* = 1.06 ± 0.04, *R*^2^ = 0.99, *P* < 0.001), but this decreased in the low-intensity samples (*β* = = 0.22 ± 0.07, *R*^2^ = 0.93, *P* < 0.001) due to the increasing randomness at the highest dilution level (Fig. 2).

### Quantification with ddPCR and two qPCR assays

Among the technical replicates, haemosporidian quantities assessed by ddPCR showed an average difference of 1.4-fold in the same reaction plate or a maximum 1.3-fold difference between reactions (*rpt* = 0.955 ± 0.008, *P* < 0.001). The intra-reaction variation in Cq values ranged between 0.01 and 1.22 for general qPCR, which is equal to a 2.3-fold difference (77% lower consistency than ddPCR) in haemosporidian quantities (*rpt* = 0.902 ± 0.02, *P* < 0.001), although the obtained amplification efficiencies in all qPCR experiments were approximately the same (91 ± 0.02%). Inconsistencies between replicates (i.e. one positive while the other one or two negative) in ddPCR (98.5%) was also higher than in the general qPCR (94%).

**Fig. 3:**
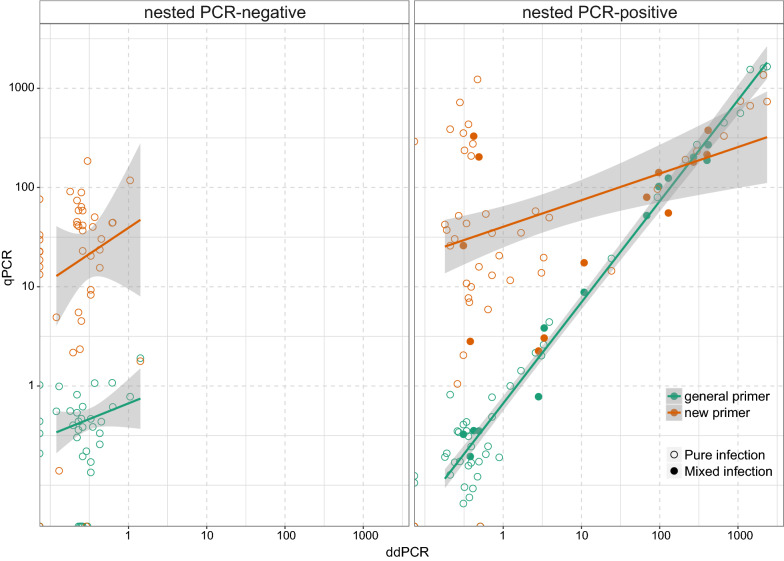
Correlation between qPCR and ddPCR results. Each dot represents one sample. *Note*: Both x-axis and y-axis were log_10_-converted

Haemosporidian quantities assessed by ddPCR and general qPCR were significantly correlated with a slope close to 1 (*β* = 1.02 ± 0.03, *R*^2^ = 0.95, *P* < 0.001), ranging from approximately 1×10^−5^ (i.e. 1 copy per 10^5^ cells) to 0.3 by ddPCR (Additional file 1: Table S1). These results indicate a high consistency between these two methods. However, this correlation was dominated by the nested PCR-positive samples. When examining the two groups of samples separately (Fig. 3), the correlation between ddPCR and general qPCR results was still significant in the nested PCR-positive group (*β* = 1.03 ± 0.02, *R*^2^ = 0.97, *P* < 0.001), without any effect of parasite genera or based on whether the sample was infected by two or more lineages. For the nested PCR-negative group of samples, the correlation was no longer significant (*β* = 0.32 ± 0.21, *R*^2^ = 0.05, *P* = 0.15).

Results from the new primer qPCR also showed a positive correlation with ddPCR but with much weaker support (*β* = 1.08 ± 0.11, *R*^2^ = 0.47, *P* < 0.001), especially when haemosporidian quantities were lower than 1 copy/μl. For samples with relatively high quantities (i.e. > 1 copy/µl by ddPCR), both qPCR assays showed almost identical results (*t* = − 1.2037, *df* = 25, *P* = 0.24), whereas those with low quantities, qPCR with the new primer demonstrated significantly higher and less robust values than general qPCR (*t* = 4.115, *df* = 73, *P* < 0.001), presenting a scattered pattern.

## Discussion

In this study, we first developed a ddPCR assay for absolute quantification of avian haemosporidian parasites belonging to three genera. The method can assess haemosporidian quantities reliably from very low quantities up to relatively high levels of infection intensity (i.e. approximately 7%) without ideal laboratory-based samples or standard curves. The novel ddPCR assay yielded higher reproducible quantifications and highly consistent measurements when compared to a general qPCR assay that has been widely used in previous studies [17, 27]. This protocol can therefore serve as a robust tool for directly quantifying infection intensities of haemosporidian parasites.

When compared with previous molecular assays for haemosporidian diagnostics, we found considerable differences in detection probability. More positive samples were detected by ddPCR and qPCR than by nested PCR or light microscopy, and more than 60% (26/42) of the nested PCR-negative samples and 74% (57/77) microscopy-negative birds were identified as positive by both ddPCR and qPCR. Generally, the nested PCR-negative samples presented significantly lower infection intensities that the nested PCR-positive ones (Additional file 1: Table S1). This result supports previous studies in which ddPCR and qPCR appear to be the most sensitive methods when analysing haemosporidian parasites with low infection intensities [15]. However, quantified haemosporidian levels in nested PCR-negative samples showed poor correlation between the two assays, which cannot be simply explained by sensitivity of methods. One possible explanation could be that the number or parasites in the templates is stochastic, especially in cases of low infection intensity when the original haemosporidian quantity was close to zero. This assumption was supported by qPCR results demonstrating that several samples with low infection intensities showed disagreement between replicates (i.e. one of the replicates was positive while the other negative, data not shown).

Since all samples were collected from naturally infected birds, we cannot confidently define false positives from true infections. False positives are normally caused by non-specific amplification or primer dimer formation during a PCR reaction. The latter can be identified by checking melting curves in qPCR, removing the “rain” droplets in ddPCR, and comparing with NTCs in both methods. Given that the amount of host genomic DNA was notably higher than parasite DNA in the template, non-specific amplification may have occurred when the primer coincidentally matched a gene fragment from the host genome, which was 50 times larger than that of the parasite [28]. It was determined that the new primer (3524F-3655R) qPCR showed significantly lower correlation with quantifications of ddPCR than the general qPCR assay with primers (343F-496R) optimized to avoid non-specific amplifications (Fig. 3). This was further confirmed by electrophoresis of standard PCR samples that showed non-specific fragments were more easily amplified than in the low-intensity samples (Additional file 2: Figure S1).

The increased suppression with ddPCR of non-specific amplification compared to new primer qPCR, especially for raptors with low haemosporidian quantity, may likely contribute to the lower inhibiting effect based on the nano-litre sized reaction system in the droplets, which is one of the major advantages of ddPCR. To ensure amplification efficiency, PCR primers with relatively high GC content, and therefore higher annealing temperature, are preferred [29]. However, the overall GC content of avian haemosporidian genomes is generally very low [30], making choices narrow. Given that the host genome is 50 times larger than the parasite genome [28], the primer may have coincidentally matched a gene fragment from the host genome. The amount of host genomic DNA was notably higher than parasite DNA in the template. Given this, when matching between the host gene and primer is high enough, non-specific amplification may occur, especially when infection intensity is low. As PCR results are largely dependent on the first few cycles, random non-specific amplification at the beginning of a reaction can make a substantial impact on the result, such as was seen with the scattered quantities assessed by qPCR with the new primers in this study (Fig. 2b). Although false positives in qPCR with the SYBR Green method can be identified by inspecting melting curves, any sufficient method to estimate the ratio of non-specific to specific amplifications is still lacking. In other words, as long as non-specific amplification occurs, the quantities of target gene fragment in initial DNA obtained from Cq value will not be accurate, whereas in ddPCR, non-specific amplification is unlikely to occur simultaneously in all independent droplets, results will be less skewed in cases of low haemosporidian quantities, even if the primers in both methods are identical. Inhibitors in ddPCR can be diluted by reducing the reaction volume, while amplification efficiency can be increased at the same time [17]. Almost 20 nested PCR-positive samples were defined as negative by microscopy of blood slides all presenting low infection intensities according to ddPCR (Additional file 1: Table S1). It can be postulated that these were abortive infections, particularly for generalist parasites with wide host ranges. Although abortive infections result in dead-end of transmission, in some cases, they are likely to induce fitness decrease to hosts and sometimes even cause mortality [31]. However, studies on abortive infections are still lacking due to technical limitations. Application of this new method to further investigate abortive studies could potentially provide new insights into evolution of parasitic diseases. It is worth noting that several nested PCR-negative samples presented higher infection intensities than nested PCR-positive samples (Additional file 1: Table S1). Primers of the nested PCR protocol used to define haemosporidian lineages were designed mainly for those infecting passerines, making it less sensitive in detecting haemosporidians in other bird orders. This was also the case for the general qPCR primers, but the latter were located on a portion of rRNA in the mitochondrial genome where genes are more conserved. Previous studies have reported that in some cases parasites cannot be detected by a general nested PCR protocol [32]. In our study, the majority of identified lineages were novel. For some of these, we failed to obtain the full-length barcoding sequence (479 bp) probably due to weak binding with the primers. It is likely that some novel haemosporidian lineages with mutation sites located in the primer-binding region could not be amplified by this general nested PCR assay, and therefore, showed as negative. Moreover, the majority of the nested PCR-negative but ddPCR- and qPCR-positive samples (21 out of 26) were collected from common kestrels (*Falco tinnunculus*), which may be infected by highly specialized haemosporidians.

For the successfully sequenced samples, haemosporidian quantities assessed by qPCR and ddPCR were highly consistent, not showing preference to specific parasite genera or bias in mixed infection samples (Fig. 3b). However, mixed infections cannot be detected by either method. To further investigate the interspecific interactions in cases of mixed infections, protocols based on lineage-specific primers for quantification are still required [7].

Another concern of the ddPCR method is the higher cost compared to qPCR. The ddPCR setup, including all equipment, will cost 2–3 times more than qPCR, and the per-sample cost is approximately three times higher than qPCR for reagents, although the price has significantly decreased from approximately 5 USD in 2016 [19] to 1.5 USD currently. Therefore, when comparing relative infection intensities within a single case study of that sufficient golden samples are available, qPCR should be a more cost-effective choice. However, for research focused on the comparison of absolute quantifications reported from different laboratories or in circumstances when golden samples are not available, ddPCR will increase comparability and make it easier to accomplish these types of comparisons. In long-term studies, given that DNA may degrade during storage, the quantity of the golden sample may change, leading to biases in inter-annual analysis. In such cases, ddPCR could be a better choice.

## Conclusions

The novel ddPCR assay we have developed appears to be sensitive and reproducible in detection and quantification of avian haemosporidian parasites without requiring golden samples or standard curves. The high consistency between ddPCR and general qPCR suggests that the current ddPCR results are reliable for estimating relative haemosporidian quantity. The ability to determine absolute quantification enables comparisons in infection intensities across larger scales, i.e. monitoring annual variations in a community or investigations of the associations between widely distributed parasites and their hosts.

## Supplementary information

**Additional file 1: Table S1.** Summary of parasite infection in tested samples. Parasite lineages defined by nested PCR and sequencing, as well as parasite quantity assessed by microscopy, ddPCR and qPCR. Infection intensity was calculated based on ddPCR results and DNA concentration of template samples.

**Additional file 2: Figure S1.** Amplification results with new primers in seven samples by standard PCR. DNA marker: DL2000 (Sangon, Shanghai, China). *Abbreviation*: C, non-template control.

**Additional file 3: Figure S2.** Output of ddPCR. The sample with high haemosporidian quantity (A03), low quantity (D03), and the non-template control (C10) are presented. **a** Distribution pattern of droplets; the raindrop function close to the threshold line represents false positives caused by primer dimer or non-specific amplifications. **b** Histogram of droplets. **c** Total count of droplets in each PCR reaction well. **d** Concentration of target gene fragments in each assessed sample, calculated with the default setting of the droplet reader.

**Additional file 4: Figure S3.** Output of qPCR. Positive samples (green) and non-template controls (red) are presented. **a** Amplification plot from which Cq values can be obtained. **b** Melting curve, with peaks that appear away from target temperature representing non-specific amplification.

## Data Availability

All data generated or analysed during this study are included in this published article and its supplementary information files. All obtained cytochrome *b* sequences in this study have been deposited to GenBank with accession numbers MT281461, MT281462, MT281464-MT281466, MT281474-MT281479, MT281481-MT281484, MT281489, MT281494, MT281499, MT281500, MT281502-MT281506, MT281512, MT281514, MT281516-MT281518, MT281521, MT281522 and MT281526.

## Abbreviations

qPCR: Real-time quantitative polymerase chain reaction
ddPCR: Digital droplet polymerase chain reaction
*cytb*: Mitochondrial cytochrome *b* gene
