# Supplementary figures and images for: A new protocol for absolute quantification of haemosporidian parasites in raptors and comparison with current assays
Source: Parasit Vectors. 2020 Jul 17;13:354. doi: 10.1186/s13071-020-04195-y (PMC7368712; doi:10.1186/s13071-020-04195-y)

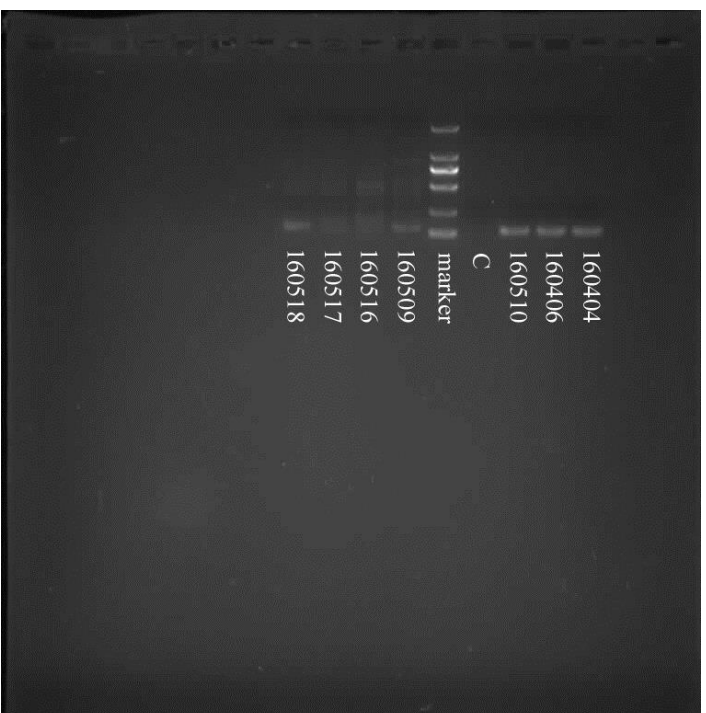

Supplement: Supplementary file 2 — Additional file 2: Figure S1. Amplification results with new primers in seven samples by standard PCR. DNA marker: DL2000 (Sangon, Shanghai, China). Abbreviation: C, non-template control. [file 13071_2020_4195_MOESM2_ESM.pdf]

a

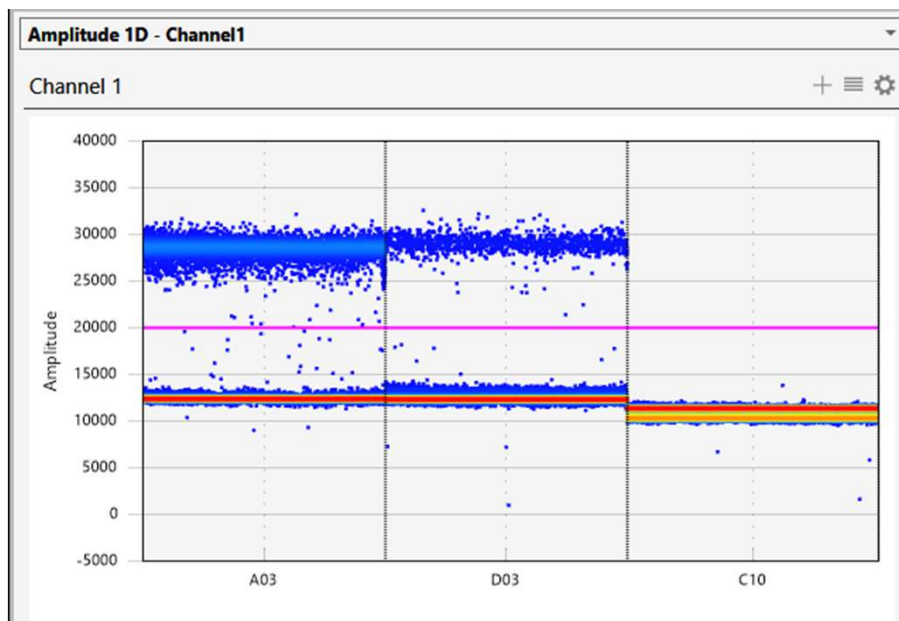

b

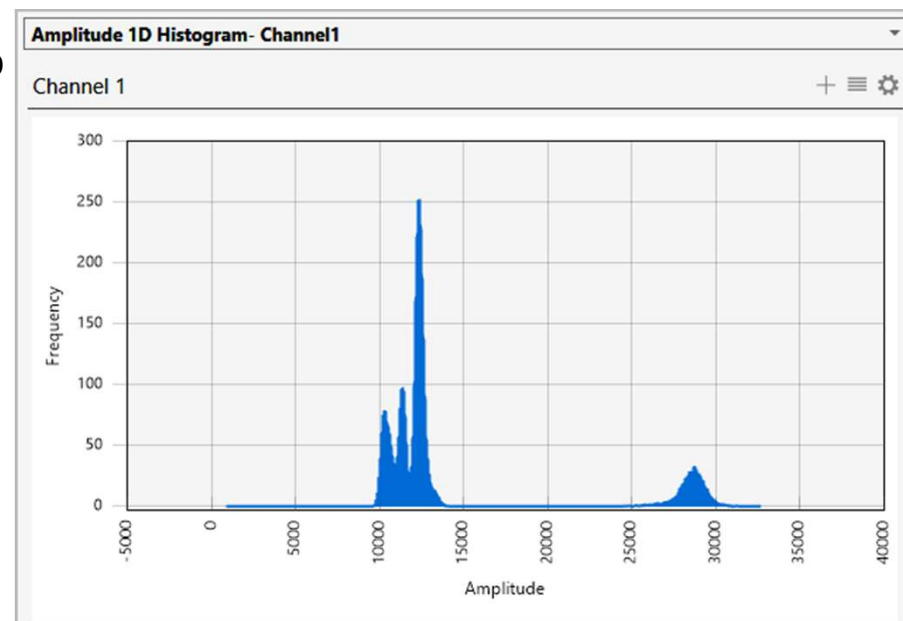

c

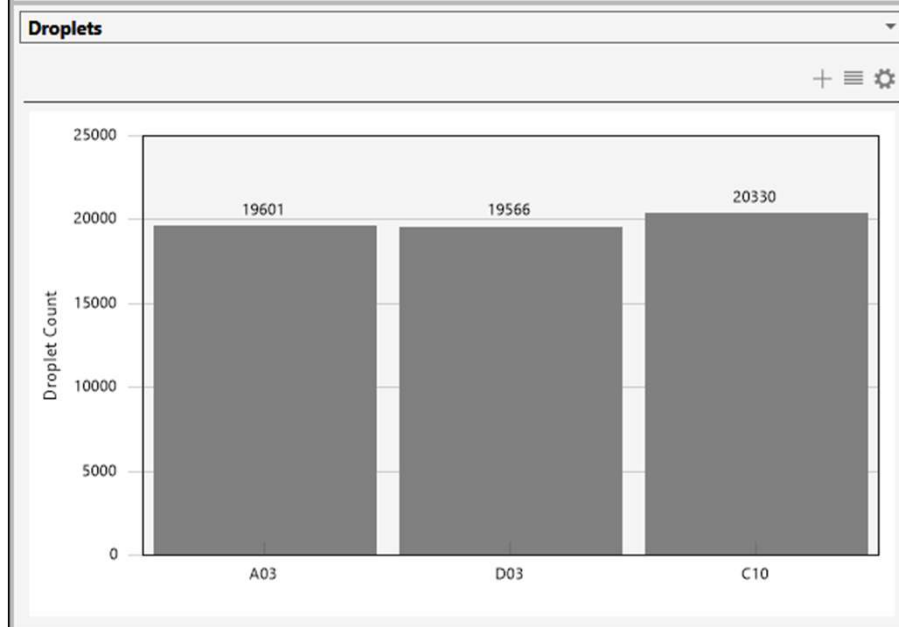

d

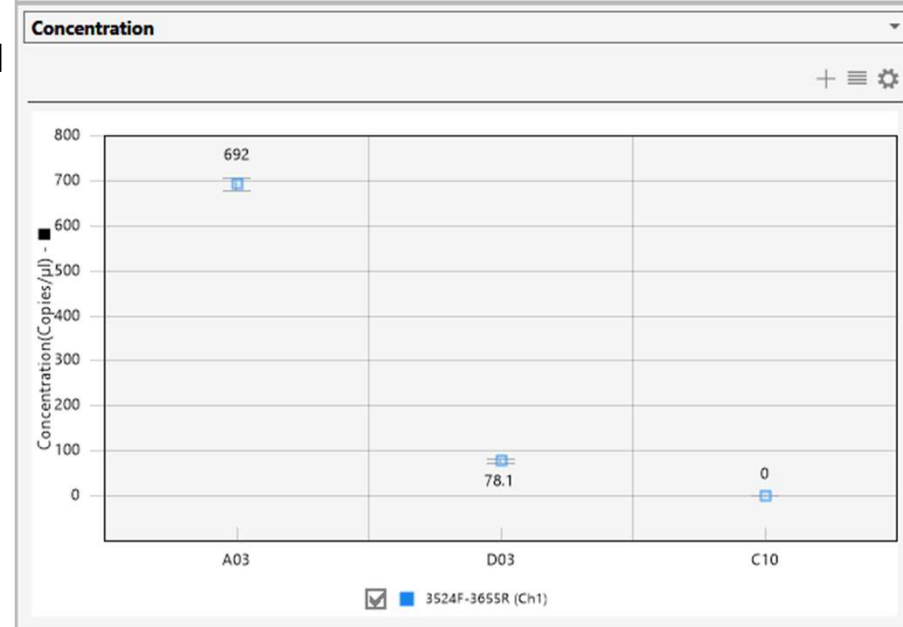

Supplement: Supplementary file 3 — Additional file 3: Figure S2. Output of ddPCR. The sample with high haemosporidian quantity (A03), low quantity (D03), and the non-template control (C10) are presented. a Distribution pattern of droplets; the raindrop function close to the threshold line represents false positives caused by primer dimer or non-specific amplifications. b Histogram of droplets. c Total count of droplets in each PCR reaction well. d Concentration of target gene fragments in each assessed sample, calculated with the default setting of the droplet reader. [file 13071_2020_4195_MOESM3_ESM.pdf]

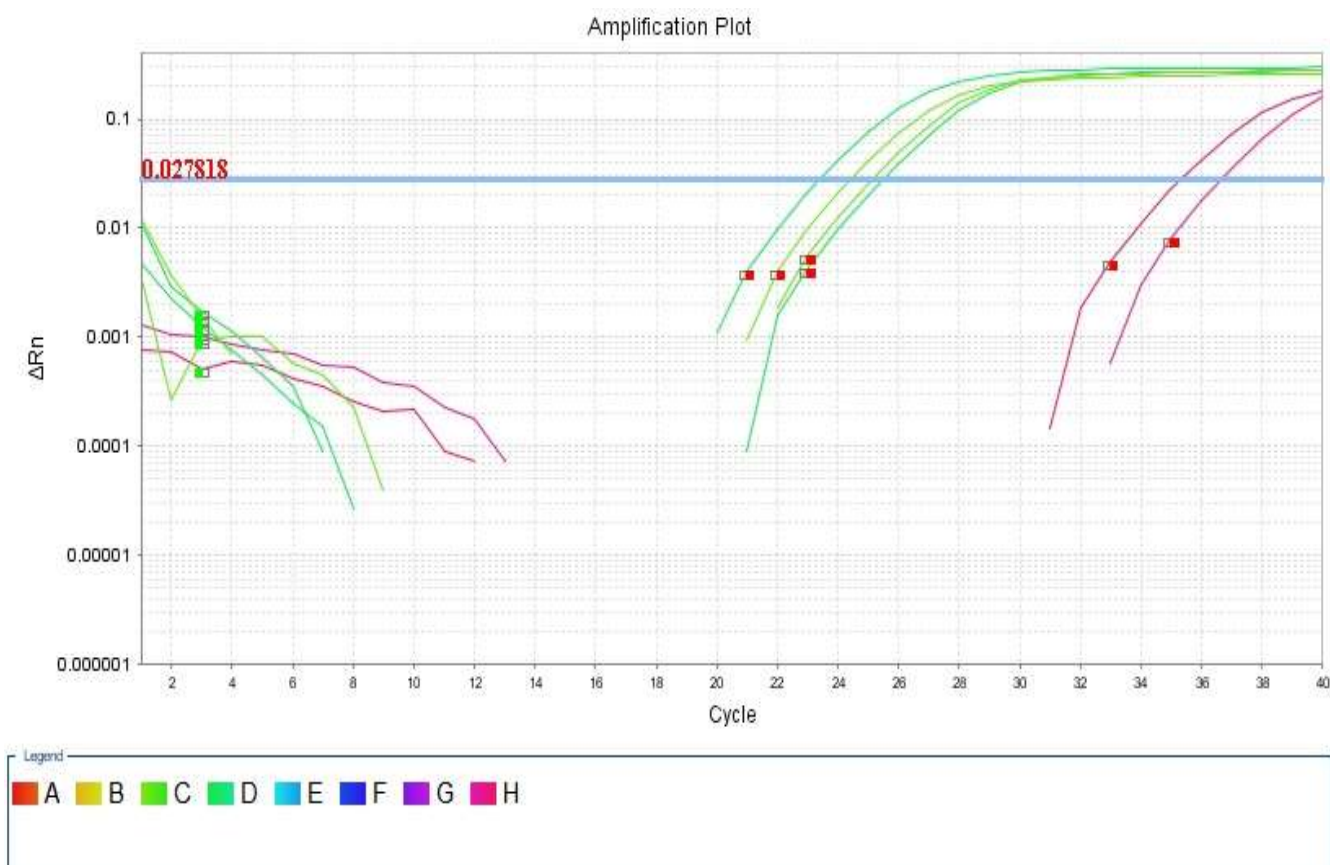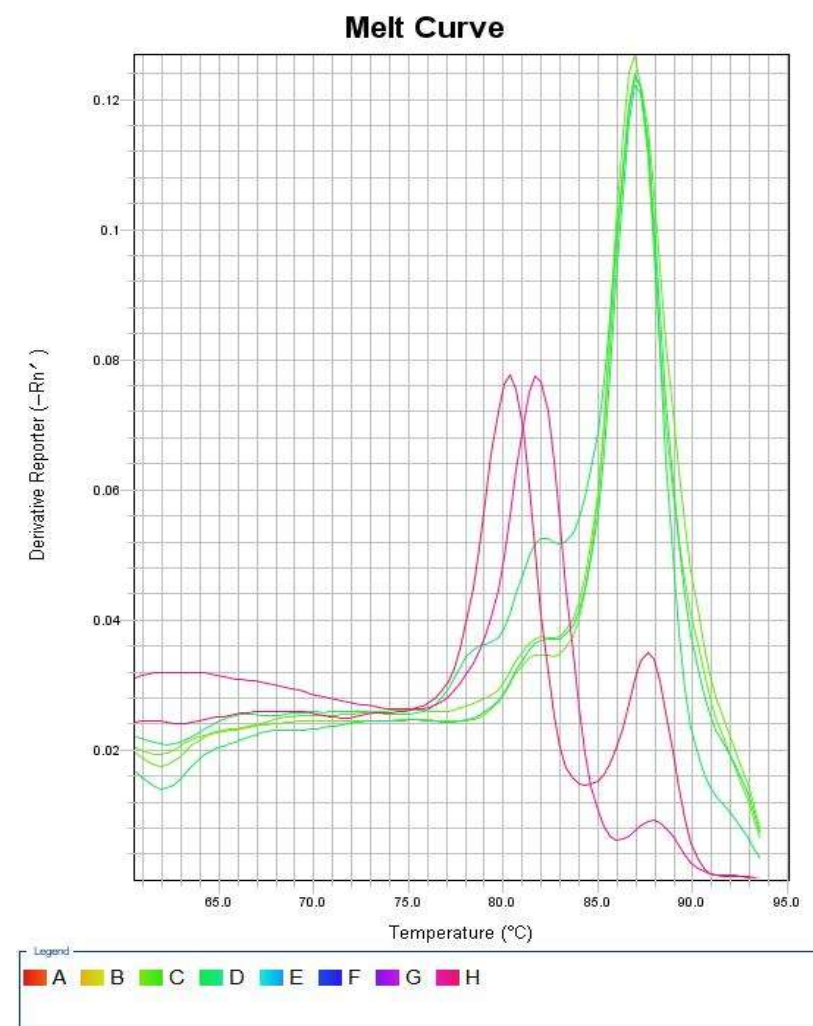

Supplement: Supplementary file 4 — Additional file 4: Figure S3. Output of qPCR. Positive samples (green) and non-template controls (red) are presented. a Amplification plot from which Cq values can be obtained. b Melting curve, with peaks that appear away from target temperature representing non-specific amplification. [file 13071_2020_4195_MOESM4_ESM.pdf]
